# Association of Adjuvant Hormone Therapy Timing With Overall Survival Among Patients With Hormone Receptor–Positive Human Epidermal Growth Factor Receptor-2–Negative Early Breast Cancer Without Chemotherapy

**DOI:** 10.1001/jamanetworkopen.2021.45934

**Published:** 2022-02-15

**Authors:** Fangmeng Fu, Liuwen Yu, Bangwei Zeng, Minyan Chen, Wenhui Guo, Lili Chen, Yuxiang Lin, Jialin Hou, Jing Li, Yan Li, Shengmei Li, Xiaobin Chen, Wenzhe Zhang, Xuan Jin, Weifeng Cai, Kun Zhang, Hanxi Chen, Yibin Qiu, Qian Nie, Chuan Wang, Lisa Jacobs

**Affiliations:** 1 Department of Breast Surgery, Fujian Medical University Union Hospital, Fuzhou, Fujian, Fujian Province, China; 2 Department of General Surgery, Fujian Medical University Union Hospital, Fuzhou, Fujian, Fujian Province, China; 3 Breast Cancer Institute, Fujian Medical University, Fuzhou, Fujian, Fujian Province, China; 4 Administration Department of Nosocomial Infection, Fujian Medical University Union Hospital, Fuzhou, Fujian Province, China; 5 Division of Surgical Oncology, Department of Surgery, Johns Hopkins School of Medicine, Baltimore, Maryland

## Abstract

**Importance:**

Studies have shown that delayed initiation of surgery and adjuvant chemotherapy is associated with lower rates of breast cancer survival. However, it remains unclear whether delayed initiation of adjuvant hormone therapy (AHT) is associated with survival.

**Objective:**

To assess the association of time to adjuvant hormone therapy (TTH) with breast cancer survival and evaluate the factors associated with AHT.

**Design, Setting, and Participants:**

This cohort study examined data from the National Cancer Database from 2004 through 2014 to assess the association of TTH (stratified as ≤150 and >150 days) with cancer survival. All patients included were diagnosed with stage I to stage III hormone receptor–positive, human epidermal growth factor receptor-2 (*ERBB2*; formerly *HER2*)–negative invasive breast cancer and underwent AHT without chemotherapy. Data were analyzed from April 2019 to May 2020.

**Exposures:**

AHT was administered at different time points following surgical procedures for breast cancer treatment.

**Main Outcomes and Measures:**

An inverse probability of treatment weighting (IPTW) model was constructed to evaluate overall survival by adjusting for treatment facility, patient demographics, tumor characteristics, and treatment; multivariable logistic regression was conducted to assess factors associated with delayed treatment.

**Results:**

A total of 144 103 patients (median [IQR] follow-up, 36.6 months [25.5-49.2 months]; mean [SD] age, 63.7 [11.6] years) were identified, which included 142 916 (99.2%) women, 11 574 (8.0%) Black patients, and 126 013 (87.4%) White patients. Of these, 134 873 patients (93.6%) had a TTH of 150 days or less and 9230 patients (6.4%) had a TTH longer than 150 days. The IPTW-based Cox model demonstrated that patients with delayed AHT (ie, a TTH past 150 days) were associated with decreased survival (hazard ratio [HR], 1.31; 95% CI, 1.26-1.35; *P* < .001) compared with those receiving the timely treatment (TTH ≤150 days). Several sensitivity analyses (including IPTW with stabilized weight [HR, 1.31; 95% CI, 1.19-1.45; *P* < .001], propensity score matching [HR, 1.41; 1.13-1.76; *P* = .002], and propensity score regression adjustment [HR, 1.29; 95% CI, 1.16-1.43; *P* < .001]) and exploratory subgroup analyses yielded similar trends. Factors associated with delayed AHT included Black racial identity (OR, 1.66; 95% CI, 1.55-1.77), nonprivate insurance (eg, no insurance: OR, 1.46; 95% CI, 1.26-1.70), living in large metropolitan or metropolitan areas (reference vs urban, less urban, or rural: OR, 0.82; 95% CI, 0.76-0.87), treatment in a community hospital (reference vs academic or research: OR, 0.91; 95% CI, 0.84-0.98), Charlson-Deyo Comorbidity Index score 2 or higher (OR, 1.17; 95% CI, 1.04-1.32), poor grade differentiation (OR, 1.42; 95% CI, 1.32-1.53), II and III pathological stage (stage III: OR, 3.13; 95% CI, 2.76-3.54), estrogen receptor–positive (ER+)/progesterone receptor–negative (PR−) or ER−/PR+ (OR, 1.22; 95% CI, 1.13-1.31), receiving breast conservation surgery (reference vs mastectomy: OR, 0.87; 95% CI, 0.79-0.94), and radiotherapy (reference vs no radiotherapy: OR, 0.56; 95% CI, 0.52-0.61).

**Conclusions and Relevance:**

The delay of the initiation of AHT past 150 days was associated with diminished survival in hormone receptor–positive, *ERBB2*-negative patients with breast cancer who did not receive chemotherapy. Efforts should be made to address factors associated with delayed treatment to improve survival.

## Key Points

**Question:**

Is delayed adjuvant hormone therapy (AHT) associated with reduced survival in patients with hormone receptor–positive breast cancer?

**Findings:**

In this cohort study of 144 103 US patients with hormone receptor–positive/human epidermal growth factor receptor-2–negative early breast cancer who did not undergo chemotherapy between 2004 and 2014, delays in the initiation of AHT more than 150 days were associated with diminished survival compared with patients who initiated therapy before 150 days.

**Meaning:**

These results suggest that efforts to avoid delayed AHT may improve survival rates for patients with hormone receptor–positive breast cancer.

## Teaser

This cohort study of a large database of US patients examines the association of delayed start of adjuvant hormone therapy with reductions in survival among patients with early breast cancer.

## Introduction

Hormone receptor (HR)–positive breast cancer is the most common subtype of breast cancer, accounting for about two-thirds of all breast malignant neoplasms.^1,2^ Among patients with HR-positive early breast cancer, hormone therapy is considered an integral treatment that reduces recurrence and mortality.^3^ Both tamoxifen and aromatase inhibitors are successfully used.^4,5^ For some patients at high risk, an extended 5 to 10 years of adjuvant hormone therapy (AHT) has been recommended.^6,7^ For some patients at low risk, treatment can be optimized by receiving HT alone without chemotherapy, thus avoiding overtreatment and eliminating the toxicity caused by chemotherapy.^8,9^

Although AHT for HR-positive early breast cancer has become a consensus treatment, the specific timing of initiating treatment remains unknown. Studies have revealed that delays in the initiation of surgical procedures or postoperative chemotherapy were associated with diminished survival.^10,11,12,13^ Recently, concerns have emerged whether delayed AHT has a similar correlation with survival. Preclinical experiments have reported that hormone therapy might affect cell cycle kinetics, inhibit tumor cell proliferation, and prevent the accelerated growth of micrometastases after primary tumor removal.^14,15,16^ A 2020 study^17^ found that patients with HR-positive breast cancer without chemotherapy experienced worse survival when initiating AHT after more than 180 days. Given that relevant studies are limited, the timing of initiation of AHT and whether delays in AHT lead to a poor prognosis need to be further explored.

To clarify the timing of initiation of AHT and the association with survival for HR-positive and *ERBB2* (formerly *HER2*)-negative early breast cancer without chemotherapy, we conducted a large population-based retrospective study using data from the National Cancer Database (NCDB). In addition, we assessed the factors associated with postponing AHT.

## Methods

The NCDB is a collaboration of the Commission on Cancer of the American College of Surgeons and the American Cancer Society. It is a hospital-based clinical cancer registry that contains approximately 70% of newly diagnosed cancer cases in the US. Patient information, including the type of treatment facility, demographics, tumor characteristics, treatment, and overall survival (OS) are recorded. This study obtained NCDB data with the approval of the Johns Hopkins Medicine institutional review board; informed consent requirements were waived because data were deidentified. Data were analyzed and reported following the Strengthening the Reporting of Observational Studies in Epidemiology (STROBE) reporting guideline.

The NCDB data was used to identify patients with stage I to III HR-positive and *ERBB2*-negative invasive early breast cancer who were diagnosed between 2004 and 2014. The study was limited to patients who underwent surgical procedures and received AHT (eFigure in the Supplement). Patients who received neoadjuvant systemic treatment, chemotherapy, immunotherapy, or had prior cancer diagnosed were excluded. We also excluded patients whose follow-up times were less than 8 months because the shorter time may not have been long enough to have received AHT and the deaths during this period were likely not related to AHT. Additionally, patients with any missing variables of interest were excluded (eTable 1 in the Supplement).

Time to adjuvant hormone therapy (TTH) was defined as the time interval from the definitive curative operation to the start of AHT. We used an NCDB variable to indicate the sequencing of systemic treatment and surgical procedure and used the number of days between diagnosis and hormone therapy and between diagnosis and surgical procedure to calculate the TTH. We used survival ROC package in R to draw the receiver operating characteristic (ROC) curve to determine the cutoff value of TTH as a continuous variable and found that near 150 days was a suitable cutoff value. Based on this determination, we divided patients into 2 groups: the timely treatment group (TTH, ≤150 days) and the delayed treatment group (TTH, >150 days). The outcome OS was defined as the number of months between the date of diagnosis and the date the patient was last contacted or died.

From the NCDB, we extracted data on age, sex (as defined by NCDB), race, insurance type, setting, facility type, Charlson-Deyo Comorbidity Index score (CCI), histology, grade of differentiation, pathological stage, hormone receptor status, surgical procedure performed, and radiotherapy. Race was stratified by White, Black, and other according to NCDB data and included as an important factor variable in study outcomes. Since the American Society of Clinical Oncology and College of American Pathologists adjusted the threshold for estrogen receptor (ER) and progesterone receptor (PR) positivity from 10% to 1% of tumor nuclei in 2010, 10% may have been used before and 1% may have been used after 2010. For detailed description of all variables, refer to the NCDB participant user file data dictionary.^18^

We compared the baseline characteristics of patients using the χ^2^ test for categorical variables and the *t* test for continuous variables. Considering the lack of randomization, we used inverse probability of treatment weighting (IPTW) to adjust for covariate differences between groups to control confounders.^19,20,21^ The IPTW approach created a weighted cohort of patients with similar measured characteristics based on propensity score (PS), which allowed the inclusion of all patients and did not require matching patients. A multivariable logistic regression model was performed to estimate PSs to obtain an unbiased average treatment effect. We assigned patients in the delayed treatment group (TTH >150 days) a weight of 1/PS and patients in the timely treatment group (TTH ≤150 days) weight of 1/(1−PS). We used standardized differences to assess the balance of covariates, which conventionally required a difference of 10% or less.

Several sensitivity analyses were conducted: (1) an IPTW model with stabilized weight to reduce variability; (2) a machine-learning algorithm from the twang package in R software to fit a generalized boosted regression model, which is superior to traditional logistic regression models^22^; (3) a Cox model of PS regression adjustment with PS as a covariate^19^; and (4) propensity score matching (PSM) using the nearest neighbor-matching with different caliper values (0.5, 0.1, 0.01, and 0.001) for 1:1 matching and 0.5 for 1:2 matching.

We also performed exploratory subgroup analyses for all variables included in the Cox model to estimate whether the observed association differed in various subgroups. The interaction analyses were subsequently applied to evaluate the heterogeneity of the treatment effects among the various subcohorts. To explore whether the delayed treatment effect was associated with ER and PR positivity, exploratory analyses were conducted regarding the positivity threshold (ie, <10% or <1%). Separate Cox models were refitted for each subgroup to determine the hazard ratios (HRs). Additionally, we also performed a multivariable logistic regression to identify factors associated with delays in AHT.

Analyses were conducted using R version 3.1.2 (R Foundation) and SAS version 9.4 (SAS Institute Inc), with 2-sided *P* < .05 considered statistically significant. Data were analyzed from April 2019 to May 2020.

## Results

### Patient Characteristics

During the 2004 to 2014 period, a total of 144 103 patients diagnosed with HR-positive and *ERBB2*-negative disease were identified, with a median (IQR) TTH of 65 days (32-104 days). Of these, 134 873 patients (93.6%) were in the timely treatment group (mean [SD] age, 63.8 [11.5] years) with a median TTH of 59 days (31-97 days), and 9230 patients (6.4%) were in the delayed treatment group (mean [SD] age, 61.7 [11.7] years) with a median TTH of 186 days (163-231 days). The cohort included 1187 (0.8%) male and 142 916 (99.2%) female patients; 11 574 patients (8.0%) were Black, and 126 013 (87.4%) were White (Table 1). There were 132 595 patients (92.0%) with ER+/PR+ and 11 508 (8.0%) with ER+/PR− or ER−/PR+ disease.

**Table 1.** Unweighted and Weighted Patient Characteristics by Time to Adjuvant Hormone Therapy (TTH)

Characteristic	Unweighted study population, No. (%)		Standardized difference, %	Weighted study population, %		Standardized difference, %
	TTH ≤150 d (n = 134 873)	TTH >150 d (n = 9230)		TTH ≤150 d	TTH >150 d	
Age, mean (SD), y	63.8 (11.5)	61.7 (11.7)	−18.3	63.7 (11.9)	64.0 (46.5)	2.7
Sex						
Men	1132 (0.8)	55 (0.6)	−2.9	0.8	0.8	−0.6
Women	133 741 (99.2)	9175 (99.4)		99.2	99.2	
Race						
Black	10 353 (7.7)	1221 (13.2)	15.2	7.8	10.2	0.3
White	118 518 (87.9)	7495 (81.2)		87.6	86.3	
Other/unknown^a^	6002 (4.5)	514 (5.6)		4.6	3.5	
Insurance type						
Private	65 389 (48.5)	4633 (50.2)	−3.9	48.6	48.2	−0.3
Medicaid	5607 (4.2)	637 (6.9)		4.2	5.5	
Medicare	59 504 (44.1)	3552 (38.5)		43.8	43.1	
Other government/unknown	2588 (1.9)	209 (2.3)		2.0	1.8	
None	1785 (1.3)	199 (2.2)		1.4	1.5	
Setting						
Large metropolitan/metropolitan	115 067 (85.3)	8141 (88.2)	8.5	85.5	85.5	0.1
Urban/less urban/rural	19 806 (14.7)	1089 (11.8)		14.5	14.5	
Facility type						
Community	14 164 (10.5)	1042 (11.3)	2.7	10.5	11.7	0
Comprehensive community	64 064 (47.5)	4054 (43.9)		47.4	44.9	
Academic/research	40 462 (30.0)	3003 (32.5)		30.1	31.7	
Other^b^	16 183 (12.0)	1131 (12.3)		12.0	11.8	
Charlson Comorbidity Index						
0	111 448 (82.6)	7586 (82.2)	1.7	82.6	83.3	−1.6
1	19 316 (14.3)	1327 (14.4)		14.3	13.6	
≥2	4109 (3.1)	317 (3.4)		3.1	3.0	
Histology						
Ductal	98 091 (72.7)	6628 (71.8)	1.6	72.7	72.6	0.2
Lobular	15 254 (11.3)	1100 (11.9)		11.3	11.4	
Other/unknown	21 528 (16.0)	1502 (16.3)		16.0	16.1	
Grade of cell differentiation						
Well-differentiated	49 012 (36.3)	2983 (32.3)	9.3	36.1	35.5	0.2
Moderate	65 415 (48.5)	4521 (49.0)		48.6	48.6	
Poor	12 404 (9.2)	1163 (12.6)		9.3	10.8	
Undifferentiated/anaplastic/unknown	8042 (6.0)	563 (6.1)		6.0	5.1	
Pathological stage						
I	102 967 (76.3)	6316 (68.4)	19.6	75.8	77.1	−2.2
II	29 460 (21.8)	2540 (27.5)		22.3	20.8	
III	2446 (1.8)	374 (4.1)		1.9	2.1	
Hormone receptor						
ER+/PR+	124 218 (92.1)	8377 (90.8)	−4.8	92.0	92.3	0.9
ER+/PR− or ER−/PR+	10 655 (7.9)	853 (9.2)		8.0	7.7	
Surgical procedure						
Breast conservation	96 992 (71.9)	7261 (78.7)	15.7	72.4	73.5	2.6
Mastectomy	37 881 (28.1)	1969 (21.3)		27.7	26.5	
Radiotherapy						
Yes	1969 (67.8)	7357 (79.7)	27.2	68.6	68.4	−0.4
No	43 375 (32.2)	1873 (20.3)		31.4	31.6	

Abbreviations: ER, estrogen receptor; PR, progesterone receptor.

^a^ Categories reported in the National Cancer Database included American Indian/Aleutian/Eskimo, Chinese, Japanese, Filipino, Hawaiian, Korean, Vietnamese, Laotian, Hmong, Kampuchean (including Khmer and Cambodian), Thai, Asian Indian/Pakistani, Pakistani, Micronesian, Chamorran, Polynesian, Tahitian, Samoan, Tongan, Melanesian, Fiji Islander, New Guinean, Other Asian, Pacific Islander, and other/unknown.

^b^ Included the Integrated Network Cancer Program and other/unknown types of cancer programs.

After using IPTW, the percentage of the overall PS standardized mean difference was reduced by 93.5%. In the unweighted cohort, we observed that patient characteristics more likely to experience delayed AHT included Black race (1221 of 11 574 patients [10.5%] vs White patients, 7495 of 126 013 [5.9%]), uninsured (199 of 1984 patients [10.0%] vs private insurance, 4633 of 70 022 [6.6%]), poorly differentiated (1163 of 13 567 patients [8.6%] vs well differentiated, 2983 of 51 995 [5.7%]), and stage II/III disease (2914 of 34 820 patients [8.4%] vs stage I, 6316 of 109 283 [5.8%]). In the weighted cohort, the covariates were well balanced with all the standardized differences of less than 10%.

### Survival Analyses

**Figure 1.**
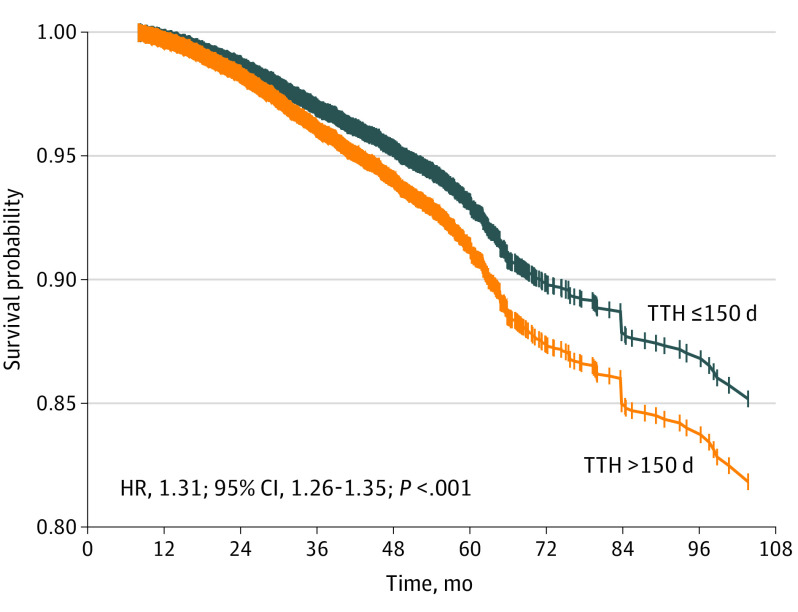
Cumulative Survival Probability Estimated Based on IPTW-Weighted Multivariate Cox Regression Model HR indicates hazard ratio; IPTW, inverse probability of treatment weighting; TTH, time to adjuvant hormone therapy.

**Figure 2.**
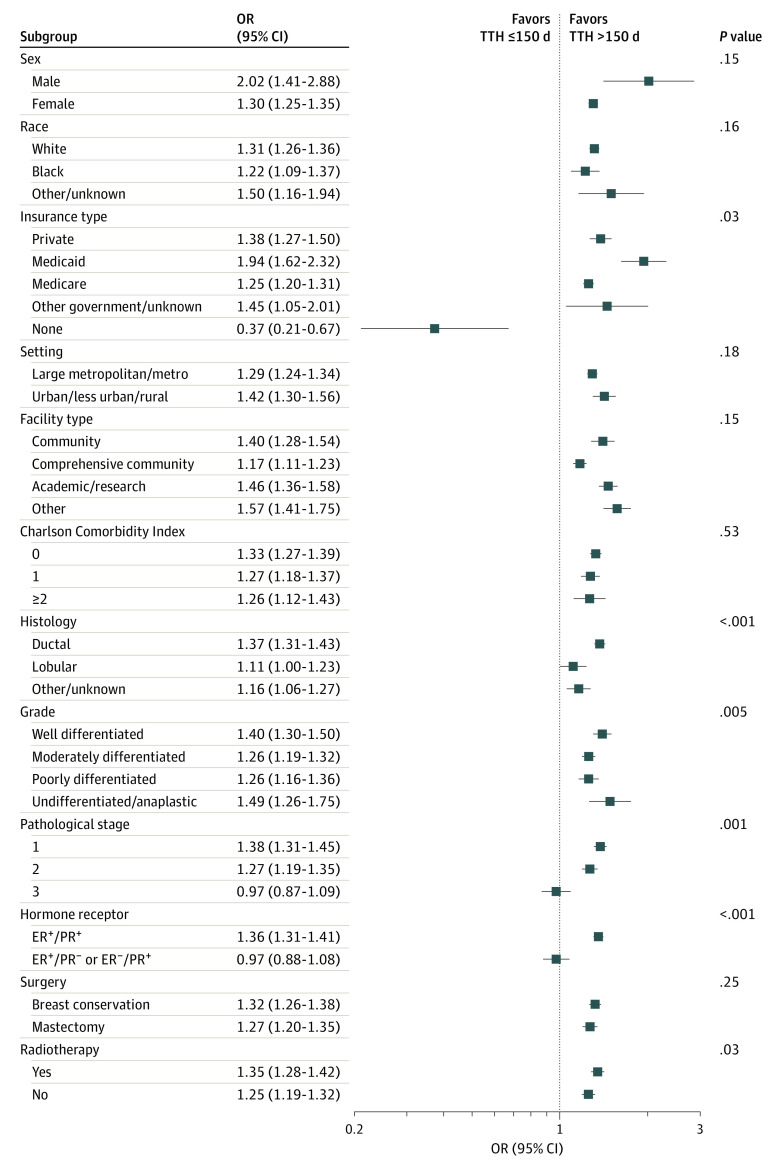
Forest Plot of the Correlation Between Delayed AHT and OS in Patient Subgroups AHT indicates adjuvant hormone therapy; ER, estrogen receptor; OR, odds ratio; OS, overall survival; PR, progesterone receptor. Other/unknown categories for race and ethnicity, as reported in the National Cancer Database, included American Indian/Aleutian/Eskimo, Chinese, Japanese, Filipino, Hawaiian, Korean, Vietnamese, Laotian, Hmong, Kampuchean (including Khmer and Cambodian), Thai, Asian Indian/Pakistani, Pakistani, Micronesian, Chamorran, Polynesian, Tahitian, Samoan, Tongan, Melanesian, Fiji Islander, New Guinean, Other Asian, Pacific Islander, and other/unknown.

The median (IQR) follow-up was 36.6 months (25.5-49.2 months). Unweighted multivariable Cox survival analysis demonstrated that patients with a TTH greater than 150 days were associated with worse survival than those with a TTH less than 150 days. Adjustments were made for age, sex, race, insurance type, setting, facility type, CCI, histology, grade of differentiation, pathological stage, hormone receptor status, surgery, and radiotherapy via IPTW. The IPTW model indicated that patients who experienced delayed treatment were associated with a 31% increase in the risk of death (hazard ratio [HR], 1.31; 95% CI, 1.26-1.35; *P* < .001) (Table 2). The estimated survival probability based on the IPTW weighted-multivariable Cox regression model is plotted in Figure 1. Exploratory subgroup analyses found a trend of OS reduction associated with delayed treatment that remained in all subgroups except for uninsured patients, pathological stage III disease, and single HR-positive (ER+PR− or ER−PR+) disease (Figure 2). The exploratory result showed that delaying treatment is associated with a worse OS among patients with pathological stage I (HR, 1.38; 95% CI, 1.31-1.45) and stage II (HR, 1.27; 95% CI, 1.19-1.35), but there was no significant correlation with pathological stage III disease (HR, 0.97; 95% CI, 0.87-1.09). A worse OS was also observed in double HR-positive disease (HR, 1.36; 95% CI, 1.31-1.41) but not in single HR-positive disease (HR, 0.97; 95% CI, 0.88-1.08) when the initiation of AHT was postponed.

**Table 2.** Multivariable Cox Proportional Hazards Model for Overall Survival

Characteristic	Hazard ratio (95% CI)	*P* value
TTH		
≤150 d	1 [Reference]	NA
>150 d	1.31 (1.26-1.35)	<.001
Age, per year	1.07 (1.07-1.08)	<.001
Sex		
Men	1 [Reference]	NA
Women	0.62 (0.54-0.71)	<.001
Race		
White	1 [Reference]	NA
Black	1.18 (1.12-1.25)	<.001
Other/unknown^a^	0.75 (0.66-0.85)	<.001
Insurance type		
Private	1 [Reference]	NA
Medicaid	2.52 (2.30-2.75)	<.001
Medicare	1.34 (1.27-1.41)	<.001
Other government/unknown	1.42 (1.22-1.66)	<.001
None	1.02 (0.80-1.31)	.85
Setting		
Large metropolitan/metropolitan	1 [Reference]	NA
Urban/less urban/rural	1.09 (1.03-1.14)	.001
Facility type		
Community	1 [Reference]	NA
Comprehensive community	0.78 (0.74-0.82)	<.001
Academic/research	0.72 (0.68-0.76)	<.001
Other^b^	0.88 (0.82-0.94)	<.001
Charlson Comorbidity Index		
0	1 [Reference]	NA
1	1.64 (1.57-1.71)	<.001
≥2	2.81 (2.64-3.00)	<.001
Histology		
Ductal	1 [Reference]	NA
Lobular	0.86 (0.81-0.91)	<.001
Other/unknown	0.85 (0.81-0.90)	<.001
Grade of cell differentiation		
Well-differentiated	1 [Reference]	NA
Moderate	1.13 (1.08-1.18)	<.001
Poor	1.74 (1.65-1.84)	<.001
Undifferentiated/anaplastic/unknown	1.31 (1.20-1.43)	<.001
Pathological stage		
1	1 [Reference]	NA
2	1.67 (1.60-1.74)	<.001
3	3.94 (3.68-4.21)	<.001
Hormone receptor		
ER+/PR+	1 [Reference]	NA
ER+/PR− or ER−/PR+	1.11 (1.05-1.17)	<.001
Surgical procedure		
Breast conservation	1 [Reference]	NA
Mastectomy	0.90 (0.86-0.95)	<.001
Radiotherapy		
Yes	1 [Reference]	NA
No	1.63 (1.56-1.71)	<.001

Abbreviations: ER, estrogen receptor; NA, not applicable; PR, progesterone receptor; TTH, time to adjuvant hormone therapy.

^a^ Categories reported in the National Cancer Database included American Indian/Aleutian/Eskimo, Chinese, Japanese, Filipino, Hawaiian, Korean, Vietnamese, Laotian, Hmong, Kampuchean (including Khmer and Cambodian), Thai, Asian Indian/Pakistani, Pakistani, Micronesian, Chamorran, Polynesian, Tahitian, Samoan, Tongan, Melanesian, Fiji Islander, New Guinean, Other Asian, Pacific Islander, and other/unknown.

^b^ Included the Integrated Network Cancer Program and other/unknown types of cancer programs.

Several sensitivity analyses yielded consistent trends (eTable 2 in the Supplement). The results of IPTW with stabilized weights (HR, 1.31; 95% CI, 1.19-1.45; *P* < .001), IPTW with a generalized boosted regression model (HR, 1.23; 95% CI, 1.18-1.27; *P* < .001), regression adjustment for PS (HR, 1.29; 95% CI, 1.16-1.43; *P* < .001), and 1:1 PSM analysis with a caliper of 0.001 (HR, 1.41; 95% CI, 1.13-1.76; *P* = .002) indicated that delayed treatment was associated with a worse OS compared with timely treatment. Similar trends were observed in the 1:1 matching with different calipers (0.5, 0.1, and 0.01) and the 1:2 matching with a caliper of 0.5. In exploratory analyses regarding the threshold of hormone receptor positivity, the IPTW results also indicated that regardless of the positivity threshold set to 1% (HR, 1.39; 95% CI, 1.32-1.46) or 10% (HR, 1.23; 95% CI, 1.16-1.30), patients who delayed TTH were associated with a shorter OS than patients with timely treatment.

Additionally, we performed multivariable logistic regression analyses to assess factors associated with delayed AHT. We observed that Black race (OR, 1.66; 95% CI, 1.55-1.77), nonprivate insurance coverage (eg, no insurance: OR, 1.46; 95% CI, 1.26-1.70), large metropolitan or metropolitan location (reference vs urban, less urban, and rural: OR, 0.82; 95% CI, 0.76-0.87), treatment received at a community hospital (reference vs academic or research: OR, 0.84; 95% CI, 0.77-0.92), CCI score of 2 or above (OR, 1.17; 95% CI, 1.04-1.32), poorer grade differentiation (OR, 1.42; 95% CI, 1.32-1.53), pathological stage II or III (stage III: OR, 3.13; 95% CI, 2.76-3.54), single HR-positive disease (ER+/PR− or ER−/PR+: OR, 1.22; 95% CI, 1.13-1.31), breast conservation operations (reference vs mastectomy: OR, 0.87; 95% CI, 0.79-0.94), and radiotherapy (reference vs no radiotherapy: OR, 0.56; 95% CI, 0.52-0.61) were associated with patients more likely to experience longer TTH (Table 3).

**Table 3.** Logistic Regression of Factors Associated With Delayed Time to Adjuvant Hormone Therapy^a^

Characteristic	Odds ratio (95% CI)	*P* value
Age, per year	0.98 (0.98-0.98)	<.001
Sex		
Men	1 [Reference]	NA
Women	1.27 (0.96-1.67)	.09
Race		
White	1 [Reference]	NA
Black	1.66 (1.55-1.77)	<.001
Other/unknown^b^	1.21 (1.10-1.33)	<.001
Insurance type		
Private	1 [Reference]	NA
Medicaid	1.43 (1.31-1.57)	<.001
Medicare	1.11 (1.05-1.18)	<.001
Other government/unknown	1.20 (1.03-1.38)	.02
None	1.46 (1.26-1.70)	<.001
Setting		
Large metropolitan/metropolitan	1 [Reference]	NA
Urban/less urban/rural	0.82 (0.76-0.87)	<.001
Facility type		
Community	1 [Reference]	NA
Comprehensive community	0.84 (0.78- 0.90)	<.001
Academic/research	0.91 (0.84-0.98)	.01
Other^c^	0.84 (0.77-0.92)	<.001
Charlson Comorbidity Index		
0	1 [Reference]	NA
1	1.05 (0.98-1.11)	.16
≥2	1.17 (1.04-1.32)	.009
Histology		
Ductal	1 [Reference]	NA
Lobular	1.06 (0.99-1.14)	.08
Other/unknown	1.05 (0.99-1.12)	.08
Grade of cell differentiation		
Well-differentiated	1 [Reference]	NA
Moderate	1.10 (1.05-1.16)	<.001
Poor	1.42 (1.32-1.53)	<.001
Undifferentiated/anaplastic/unknown	1.10 (1.00-1.21)	.04
Pathological stage		
1	1 [Reference]	NA
2	1.55 (1.47-1.63)	<.001
3	3.13 (2.76-3.54)	<.001
Hormone receptor		
ER+/PR+	1 [Reference]	NA
ER+/PR− or ER−/PR+	1.22 (1.13-1.31)	<.001
Surgical procedure		
Breast conservation	1 [Reference]	NA
Mastectomy	0.87 (0.79-0.94)	.001
Radiotherapy		
Yes	1 [Reference]	NA
No	0.56 (0.52-0.61)	<.001

Abbreviations: ER, estrogen receptor; NA, not applicable; PR, progesterone receptor.

^a^ Delayed time to therapy is defined as 150 or more days.

^b^ Categories reported in the National Cancer Database included American Indian/Aleutian/Eskimo, Chinese, Japanese, Filipino, Hawaiian, Korean, Vietnamese, Laotian, Hmong, Kampuchean (including Khmer and Cambodian), Thai, Asian Indian/Pakistani, Pakistani, Micronesian, Chamorran, Polynesian, Tahitian, Samoan, Tongan, Melanesian, Fiji Islander, New Guinean, Other Asian, Pacific Islander, and other/unknown.

^c^ Included the Integrated Network Cancer Program and other/unknown types of cancer programs.

## Discussion

In this NCDB cohort study, 6.4% of patients with HR-positive, *ERBB2*-negative breast cancer experienced a delay in initiating AHT longer than 150 days, which was significantly associated with poorer survival. We also determined several factors associated with delays in initiating AHT. Regardless of what factors led to delayed AHT, we observed a correlation between delayed treatment and reduced survival. The data fully demonstrated the importance of avoiding delays in treatment. The benefits of timely treatment may even be comparable with adding standard therapies, such as chemotherapy and HT.

The timing of surgical procedures and chemotherapy and how it may factor into survival is a common concern for patients with breast cancer, and it is also a question often raised when patients consult with their surgeon. Recently, several studies^10,11,12,13^ have assessed the timing of surgery and chemotherapy, but the timing of AHT remains unclear. Clinically, AHT is usually added before, simultaneously, or after radiotherapy according to the preferences of doctors and patients. Our study provides clinicians with data on the timing of AHT and demonstrates that delaying AHT was similarly associated with survival rates as delaying surgery and chemotherapy.

Lee et al^23^ showed that, compared with the current recommendation of AHT within 12 months of diagnosis, the initiation of AHT at 12 to 24 months after diagnosis may not be related to reduced survival of patients with stage II and III, HR-positive, *ERBB2*-negative breast cancer who received chemotherapy. This finding would contradict our results. However, our study aimed to determine the association of AHT timing with the survival of patients with HR-positive, *ERBB2*-negative breast cancer who received AHT but did not receive chemotherapy, and was intended to examine the optimal initiation timing of AHT within the recommended 12 months. Therefore, these 2 studies focus on different aspects of HR-positive breast cancer. Similar to our study, Li et al^17^ demonstrated that delayed AHT (>180 days) was associated with diminished survival among female patients with HR-positive disease who were not treated with chemotherapy. However, *ERBB2* status was not considered, and the number of cases and the baseline characteristics varied significantly between the groups, which created a non-negligible confounding bias. In our study, we excluded the effect of *ERBB2*-positive disease and performed IPTW methods to control for confounding bias. The differences in cutoff points may be due to differences in the enrolled populations.

Tumor grade and disease stage are closely related to the risk of recurrence and death from breast cancer. We found that pathological stage II or III disease and less differentiated tumors (ie, moderately, poorly, undifferentiated, anaplastic, or unknown) appear to be critical factors associated with the likelihood of postponing AHT. Previous studies have also reported that patients with advanced-stage breast cancer have higher percentages of delayed or refused treatment and greater risk of death associated with delay increases.^24,25^ The reasoning behind patients with stage III disease (2%) in our cohort who did not get chemotherapy is not clear, but regardless of the reason, after adjusting for confounders via the IPTW model, our exploratory subgroup analyses found that delayed AHT was only associated with the OS of stage I or II diseases, but not with stage III disease. Moreover, interaction analyses showed significant differences between the subgroups. Given the poor prognosis and high baseline mortality of patients with stage III or less-differentiated disease, the association with survival seemed to be slight when delayed AHT occurred. It is worth noting that our analyses were limited to patients who had not received chemotherapy, but the effect of delayed AHT on stage III patients receiving chemotherapy remains unknown, and further study is needed.

This study demonstrated that patients with single HR-positive breast cancer are more likely to have delayed AHT and have a poorer prognosis than double HR-positive patients, which is consistent with the results of other studies.^26,27^ In exploratory subgroup analyses, we found that delaying AHT was associated with reduced OS in patients with double HR-positive disease, but not with reduced OS for patients with single HR-positive disease. The interaction analysis also showed significant differences between the 2 subgroups. This may also be explained by the poor prognosis of single-positive disease, which would mask the influence of delayed AHT on survival. Therefore, for patients with double-positive, stage I or II diseases, the treatment decision time should be shortened as much as possible to improve the outcome.

An association between delayed treatment with breast-conserving surgical procedures and radiotherapy was also observed in this study. This delay may be due to postoperative complications, long waiting times, or the treatment duration of radiotherapy. However, the OS of patients receiving radiotherapy was better than patients without radiotherapy, which suggests that the survival benefits from radiotherapy overshadow the impact of delaying AHT for months. The combination of HT and radiotherapy may improve the local control rate by enhancing cytotoxicity and improving the tumor response, but it also raises the risk of lung fibrosis.^28,29,30^ Thus, the optimal sequence of postoperative HT and radiotherapy remains unresolved, and should be explored in future studies to maximize efficacy and achieve the lowest toxicity. Regardless of the sequence, both therapies should be performed as soon as possible postoperation to avoid the adverse outcomes associated with delay.

We found that the likelihood of delaying AHT is higher among younger patients, which may be explained by concerns of adverse reactions and fertility issues,^31^ or because young patients are more likely to undergo mastectomy and breast reconstruction surgery.^32,33^ Although the risk of delayed treatment is lower in older patients than for younger patients, the OS of older patients is worse. This may be related to undertreatment^34^ or poor tolerance, compliance, and discontinuing therapy.^35,36^ Furthermore, it should also be noted that older patients have a higher risk of dying from other causes. However, the NCDB contains only OS data and lacks tumor-specific survival data.

This study also found that CCI scores of 2 or higher, Black race, nonprivate insurance, and treatment received in community hospitals are factors in AHT timing and thus associated with worse survival. A longer treatment decision-making process, more treatment-related complications, and an extended recovery period may be found among patients with a higher CCI. Compared with White patients, Black patients have a higher risk of delayed disease diagnosis and treatment, underuse of appropriate therapy, nonadherence, and early termination of treatment.^37,38,39^ In some populations, worse socioeconomic conditions and care resources may lead to differences in cancer screening, treatment options, and disease care.^39,40^ As for facility type, the reason for the increased survival rate of patients treated in large hospitals or teaching hospitals is not known. Community hospitals should be encouraged to enhance cancer screening and early cancer diagnosis procedures, and to improve treatment strategies to optimize patient prognosis.

### Limitations

This study had several limitations. Although we tried to minimize the inherent selection bias through the IPTW model, the confounding from unmeasured variables may remain and have affected results. Then there are the limits of the NCDB to consider. The database only provides data about the first course of treatment, and it reports OS without other survival outcomes such as cancer-specific survival or local or distant recurrence. The NCDB lacks information about the specific type, sequence, duration, adherence, and adverse reactions of HT, all of which are factors that may influence treatment decisions. The possibility of underreported factors exists because outpatient treatments are not always reported in hospital records, and the possibility of misclassification based on chart abstraction also exists. Though we cannot directly investigate these issues, it is inevitably part of all database-based analyses. Finally, doctors’ suggestions and patient preferences in the physician-patient communication process may also play a role in treatment decisions, and were not considered as part of our study.

## Conclusions

In this cohort study, the delay of the initiation of AHT past 150 days in patients with HR-positive, *ERBB2*-negative breast cancer who did not receive chemotherapy was associated with diminished survival. All patients should start AHT as soon as possible after surgical procedures and reduce unnecessary delays. Better efforts to understand these delayed treatment barriers should be a priority in the clinic to provide timelier care to patients and improve their outcomes.

## Notes

Supplement. **eTable 1.** Missing Variables Data **eTable 2.** Sensitivity Analyses Regarding the Association of TTH >150 Versus TTH ≤150 with OS **eFigure.** Flow Diagram of Exclusions for National Cancer Database (NCDB)
