# Supplementary material for: Association of Adjuvant Hormone Therapy Timing With Overall Survival Among Patients With Hormone Receptor–Positive Human Epidermal Growth Factor Receptor-2–Negative Early Breast Cancer Without Chemotherapy
Source: JAMA Netw Open. 2022 Feb 15;5(2):e2145934. doi: 10.1001/jamanetworkopen.2021.45934 (PMC8848199; doi:10.1001/jamanetworkopen.2021.45934)
Supplement: Supplement. — eTable 1. Missing Variables Data eTable 2. Sensitivity Analyses Regarding the Association of TTH >150 Versus TTH ≤150 with OS eFigure. Flow Diagram of Exclusions for National Cancer Database (NCDB) [file jamanetwopen-e2145934-s001.pdf]

Supplemental Online Content

Fu F, Yu L, Zeng B, et al. Association of adjuvant hormone therapy timing with overall survival among patients with hormone receptor–positive human epidermal growth factor receptor-2–negative early breast cancer without chemotherapy. *JAMA Netw Open.* 2022;5(2):e2145934.
doi:10.1001/jamanetworkopen.2021.45934

eTable 1. Missing Variables Data

eTable 2. Sensitivity Analyses Regarding the Association of TTH >150 Versus TTH ≤150 with OS

eFigure. Flow Diagram of Exclusions for National Cancer Database (NCDB)

This supplemental material has been provided by the authors to give readers additional information about their work.

© 2022 Fu F et al. *JAMA Network Open.*

eTable 1. Missing Variables Data.

Variable	NCDB Missing (n)*
Age	0
Sex	0
Race	0
Insurance type	0
Setting	3757
Facility type	0
Charlson Comorbidity Index (CCI)	0
Histology	0
Grade	0
Pathological stage	0
Hormone receptor	0
Surgery	29
Radiotherapy	310
*Not exclusive: some patients may have had more than one variable missing.	

© 2022 Fu F et al. *JAMA Network Open.*

eTable 2. Sensitivity Analyses Regarding the Association of TTH>150 Versus TTH≤150 with OS

**Model**	**Hazard Ratio (95% CI)**	***P* Value**
Unweighted	1.32 (1.19-1.47)	＜.001
Propensity score based		
Weighted (IPTW)	1.31 (1.26-1.35)	＜.001
IPTW with stabilized weight	1.31 (1.19-1.45)	＜.001
IPTW with a generalized boosted regression model	1.23 (1.18-1.27)	＜.001
Propensity Score Matching (PSM)[a]	1.41 (1.13-1.76)	.002
PS regression adjustment	1.29 (1.16-1.43)	＜.001

Abbreviations: IPTW, inverse probability of treatment weighting; OS, overall survival; PS, propensity score.

[a] Propensity score matching (PSM) was conducted using the nearest neighbour-matching with different calipers values (0.5, 0.1, 0.01, 0.001) for 1:1 matching and 0.5 for 1:2 matching. All results are stable. The result of a 1:1 match with a caliper of 0.001 was shown here.

© 2022 Fu F et al. *JAMA Network Open.*

eFigure 1. Flow Diagram of Exclusions for National Cancer Database (NCDB)

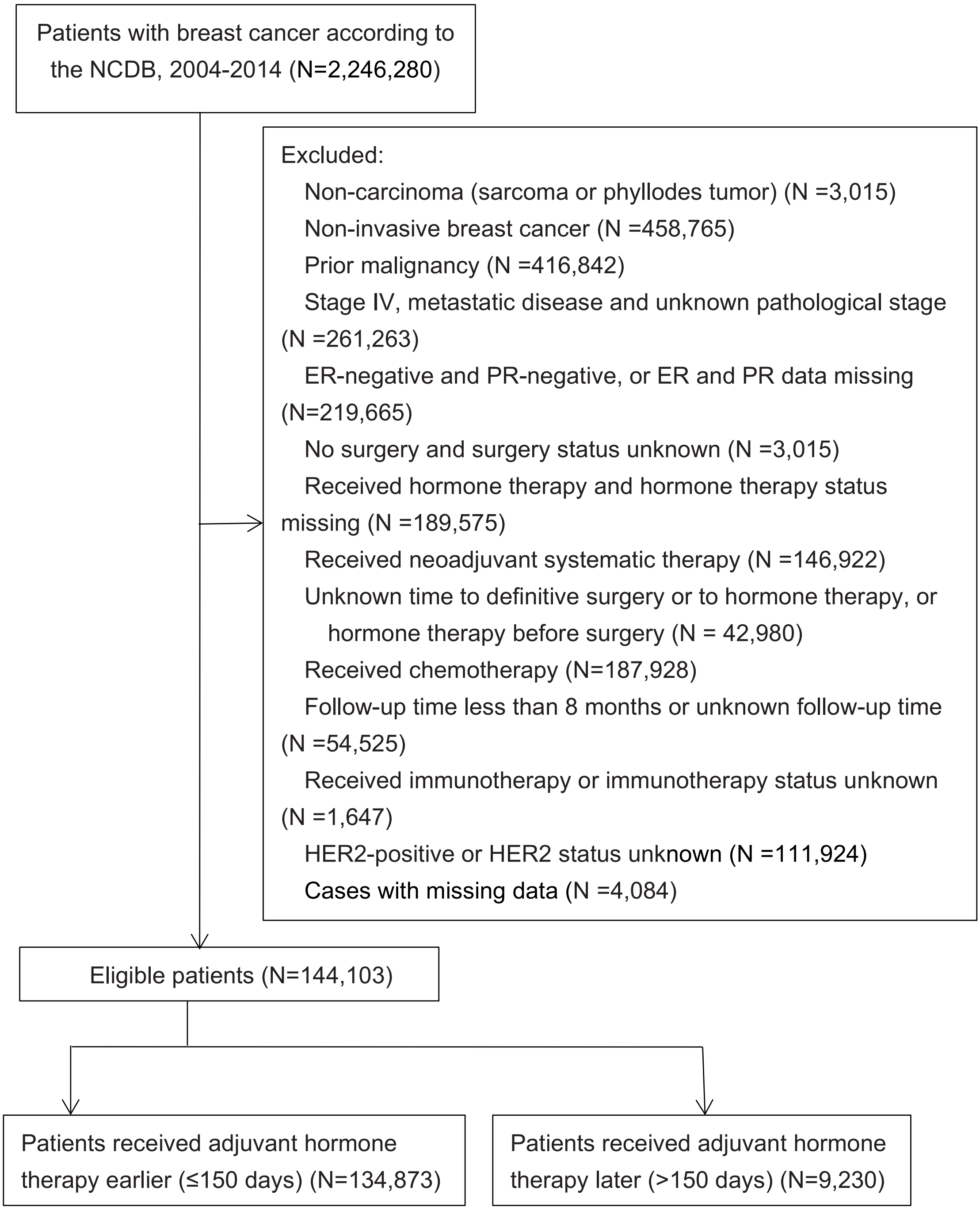

© 2022 Fu F et al. *JAMA Network Open.*
